# Transcatheter Repair of Tricuspid Valve Regurgitation: A Systematic Review

**DOI:** 10.3390/jcm13216531

**Published:** 2024-10-30

**Authors:** Aswin Srinivasan, Jonathan Brown, Alexander Rhodes, Sobia Khan, Viswanath Chinta, Pranav Loyalka, Arnav Kumar

**Affiliations:** 1 Department of Cardiology, HCA Houston Healthcare Medical Center, Tilman J. Fertitta Family College of Medicine, The University of Houston, Houston, TX 77004, USA; 2 Department of Internal Medicine, HCA Houston Healthcare Clear Lake, Tilman J. Fertitta Family College of Medicine, The University of Houston, Houston, TX 77004, USA; 3 Structural Heart & Valve Center, Houston Heart, HCA Houston Healthcare Medical Center, Tilman J. Fertitta Family College of Medicine, The University of Houston, Houston, TX 77004, USA

**Keywords:** tricuspid regurgitation, transcatheter repair, TriClip, MitraClip, PASCAL

## Abstract

**Introduction:** Clinically significant severe tricuspid regurgitation (TR) is a common untreated pathology associated with increased mortality. Even though surgical valve replacement has been the mainstay option, transcatheter intervention is a novel and potentially effective tool. To the best of our knowledge, this is the first systematic review that assessed and compared clinical and echocardiographic outcomes of coaptation and annuloplasty devices in patients with clinically significant TR. **Methods:** PubMed, the Cochrane Central Register of Controlled Trials, and EMBASE were searched for articles published from August 2016 until February 2023. Primary endpoints were technical and procedural successes. Secondary endpoints were TR grade, NYHA, change in 6 min walk distance (6MWD), and echocardiographic parameters at 30-day follow-up. **Results:** We included thirty-eight studies consisting of 2273 patients with severe symptomatic TR (NYHA III-IV 77% and severe/massive/torrential TR 83.3%) and high surgical risk (mean EUROSCORE of 7.54). The technical success for the annuloplasty devices was 96.7% and for the coaptation device was 94.8%. The procedural success for the annuloplasty devices was 64.6% and for the coaptation device was 81.4%. The 6MWD increased by 17 m for the coaptation devices and increased by 44 m after 30 days for the annuloplasty devices. A reduction in TR grade to <2 was seen in 70% of patients with coaptation and 59% of patients with annuloplasty devices. **Conclusions:** Transcatheter tricuspid valve intervention appears to be feasible and is associated with favorable outcomes.

## 1. Background

Severe tricuspid regurgitation (TR) has been associated with poor long-term outcomes, including an increase in incidences of mortality, end-organ damage, and right heart failure [1]. The tricuspid valve (TV) is the largest valve with an area between 7 and 9 cm^2^ and is located between the right atrium (RA) and right ventricle (RV) [2,3]. It is also the valve located most anteriorly. The normal TV complex contains the valve leaflets, papillary muscles, chordae tendineae, fibrous annulus, and the right ventricle and right atrial myocardium [1,4]. An increase in all-cause mortality has been noted independent of left ventricular ejection fraction (LVEF), right ventricle (RV) dysfunction, and pulmonary pressures in patients with moderate to severe TR [4,5,6].

TR is then categorized into Primary and Secondary or “Functional TR”. Primary TR is present in 8–10% of TR cases and is due to a congenital, acquired pathology of the valve or iatrogenic causes such as cardiac implantable electronic devices (CIED). Secondary TR is the most common form, accounting for the remaining 90% of cases, and is usually due to left-sided heart failure, right sided pathology, or atrial fibrillation [1,4,7]. Patients with significant TR will present with right heart failure symptoms such as fatigue, peripheral edema, ascites, and painful hepatosplenomegaly [8]. Medical, surgical, and percutaneous valve interventions are the treatment options available in the arsenal. Medical management is often challenging and focuses on symptomatic relief. Surgical valve replacements or repairs are associated with high rates of complications and 10% in-hospital mortality. Complications also include up to 34% of patients requiring permanent pacemaker placement and 5% of patients developing acute kidney injury requiring hemodialysis [9,10].

Despite these complications, surgical valve replacement is still considered first-line therapy. Transcatheter tricuspid valve intervention (TTVI) offers an attractive alternative to surgery, especially in high risk and inoperable cases. Furthermore, TTVI has been associated with lower all-cause mortality and rehospitalization when compared to optimal guideline-directed medical therapy alone [11]. In addition, TTVI is considered less invasive and safer in high-risk patients. Transcatheter tricuspid valve interventions can be classified into annuloplasty devices, replacement devices, caval valve implantation (CAVI), and coaptation devices. We aimed to qualitatively and quantitatively present the current available evidence on the safety and efficacy of annuloplasty and coaptation devices available for the treatment of tricuspid regurgitation. To our knowledge, this is the first systematic review that assessed and compared clinical and echocardiographic outcomes of coaptation and annuloplasty devices for the treatment of tricuspid regurgitation. Methods

### 1.1. Literature Search

**Figure 1:**
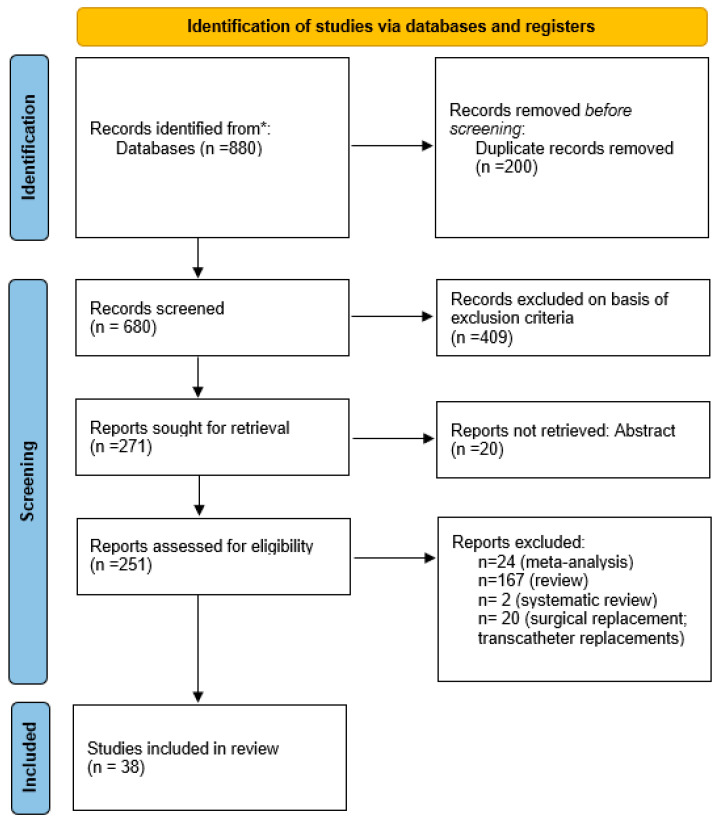
Study selection is reported according to the Preferred Recording Items for Systematic Reviews and Meta-analysis guidelines (PRISMA). * Databases: PubMed, the Cochrane Central Register of Controlled Trials, and EMBASE.

Studies were assessed using the Preferred Reporting Items for Systematic Reviews and Meta-Analyses (PRISMA) guidelines for systematic review analysis (Figure 1). Original published studies were searched in electronic databases including PubMed, the Cochrane Central Register of Controlled Trials, and EMBASE from August 2016 until August 2024. The terms used in the search criteria were ‘Transcatheter tricuspid repair’ AND ‘Annuloplasty device’ OR ‘Coaptation devices’. No patients were directly involved in the conduct of this systematic review and no informed consent or institutional review board approval was required.

### 1.2. Inclusion and Exclusion Criteria

Inclusion criteria were (i) human studies, (ii) studies presenting data on patients with primary, secondary, or mixed tricuspid regurgitation who underwent tricuspid valve repair with the TriClip, MitraClip, PASCAL, EVOQUE, FORMA, TriAlign, Cardioband, and GATE system devices (Table 1 and Table S1), and (ii) studies with at least fifteen patients. Exclusion criteria were review articles, abstracts, pre-print studies, non-research letters, viewpoints, editorials, and commentaries.

### 1.3. Data Extraction and Quality Assessment

Two reviewers (AS and JB) independently reviewed studies from August 2016 to February 2023 and extracted data on methodology, quality criteria, and outcomes. Discrepancies and disagreements were resolved with the help of a third reviewer (AR).

### 1.4. Outcome of Interest

Efficacy, safety, and clinical endpoints were the primary outcome measures. Efficacy endpoints included technical success and procedural success. Technical success, measured at the exit of the catheterization laboratory, was defined by the following: (I) an absence of procedural mortality, (II) the successful access, delivery, and retrieval of the device, (III) the successful deployment and correct positioning of the intended implant, and (IV) freedom from emergency surgery or reintervention. Clinical endpoints included change in New York Heart Association (NYHA) classification and change in mean 6 min walking distance (M6WD), TR grade reduction, and echocardiographic endpoints such as effective regurgitant orifice area (EROA), regurgitant volume, left ventricular ejection fraction (LVEF), right atrial volume, tricuspid annular plane systolic excursion (TAPSE), and vena contracta width. Safety endpoints were defined by the absence of 30 day major adverse events (MAEs), which included death, stroke, life threatening bleeding, major vascular complications, major cardiac structural complications, acute kidney injury with new dialysis, myocardial ischemia, and severe hypotension, heart failure, or respiratory failure requiring intravenous vasopressors or mechanical heart failure treatments.

The following demographic data were extracted (Table 2 and Table S2): the name and time of the study, total number of patients, number of patients with primary, secondary, or mixed MR, median age, and number of patients with atrial fibrillation, coronary artery bypass graft, cardiac surgery, COPD, coronary artery disease (CAD), diabetes, myocardial infarction, hypertension, chronic kidney disease, and stroke. The following pre-intervention data were extracted: logistic European System for Cardiac Operative Risk Evaluation (EuroSCORE), the Society of Thoracic Surgery (STS) risk score, pre-procedure NYHA class, TR grade, and pre-procedure left-ventricular ejection fraction (LVEF). Appropriate candidates for transcatheter tricuspid repair were determined by the local interdisciplinary heart team. The following factors were used to define high surgical risk: severe left ventricular dysfunction, logistic EuroSCORE > 8.11%, extensive comorbidities, and frailty. 

The following leaflet coaptation and annuloplasty devices were used in studies for transcatheter valve repair. The leaflet coaptation devices included are TriClip, PASCAL, MitraClip, and FORMA. The annuloplasty devices included are TriAlign (direct suture), TriCinch, and Cardioband (ring annuloplasty). Navigate, EVOQUE (stented), and TricValve are the valves included that were used for transcatheter valve replacement. 

## 2. Results

### 2.1. Study Selection

Studies were assessed using the Preferred Reporting Items for Systematic Review and Meta-analysis (PRISMA) guidelines for systematic reviews (Figure 1). The initial search strategy identified 880 records. After removing duplicate articles (*n* = 200), 680 studies were left and screened by title and abstract. We identified 231 articles for full-text review. Of these, 38 articles fulfilled the eligibility criteria and were included in the analysis. 

### 2.2. Study Characteristics

Baseline characteristics of patients who underwent TTVI are described in Table 2. The mean age of patients was 77 years and 58.4% were female. Most patients (77.2%) were in NYHA class III or IV. The mean Society of Thoracic Surgery (STS) score was 5.94% and the mean EUROSCORE II was 8.11. The mean 6 min walk test was 230.7 m. Comorbidities included atrial fibrillation (85.43%), a history of coronary artery bypass grafting (20.28%), prior valvular intervention (27.98%), chronic obstructive pulmonary disease (20.47%), coronary artery disease (32.89%), diabetes mellitus (25.96%), hypertension (77.57%), prior myocardial infarction (14.79%), prior stroke (21.29%), and chronic kidney disease (36.89%). Most patients had functional TR (92.4%). A total of 80 patients (4.3%) were diagnosed with degenerative TR and 61 patients (3.3%) had mixed TR. Baseline echocardiographic characteristics are summarized in Table 3. A total of 83.8% of patients had severe, massive, or torrential TR. Baseline mean effective regurgitant orifice area (EROA) was 0.77 cm^2^. Baseline mean left ventricular ejection fraction (LVEF) was 51.8%.

### 2.3. Procedure Success Rates and Short- and Intermediate-Term Outcomes

Table 4 summarizes post-procedural outcomes with weighted averages for the included studies. Technical success across all device types was 94.9%. The weighted average procedural success was 78.9%. Studies using annuloplasty reported the highest procedural success of 96.7%. The weighted average technical success was 78.9%. In studies using coaptation devices, 84.0% had a TR grade reduction by ≥1 at discharge. A total of 73.8% had a TR grade reduction by ≥1 at 30 days. A total of 72.4% had a TR grade ≤ 2 at 30 days. Functional status improved with 32.1% of patients in NYHA III/IV at 30 days across all device types. The average 6MWD improved to 250.1 m at the 30 d follow-up. Average EROA improved to 0.47 cm at the 30-day follow-up. 

### 2.4. Safety Outcomes

TR is categorized into Primary and Secondary or “Functional TR”. Primary TR is present in 8–10% of TR cases and is due to any congenital or acquired pathology of the valve such as CIED-related TR or TR related to its supporting apparatus. Secondary TR is the most common form, accounting for the remaining 90% of cases, and is usually due to left-sided heart failure or atrial fibrillation [1,4,7]. Atrial TR is due to primary annular or atrial remodeling such as atrial fibrillation. Patients with significant TR will present with right heart failure symptoms such as fatigue, peripheral edema, ascites, and painful hepatosplenomegaly [8].

Recorded outcomes regarding major adverse events (MAEs) are included in Table 5 and consist of the incidence of major bleeding, device detachment, device embolization, device thrombosis, stroke, acute kidney injury, access-site complications, conversion to open heart surgery, and in-hospital mortality. Procedural time across all studies was an average of 141.66 min with individual reported values included in Table 5. Of the 38 studies included in this review, there was a reported in-hospital mortality of 32 across all studies with a mean of 1.41%. Conversion to open heart surgery across all studies was seen in twelve patients and access-site complications were seen in nine patients. Additional 30-day MAEs are summarized. Respectively, the incidences across all studies and ranges were as follows: major bleeding (67; 0–23.8%), device detachment (18; 0–12.9%), device embolization (0; 0%), device thrombosis (1; 0–5.3%), stroke (9; 0–5.3%), and acute kidney injury (27; 0–15%).

## 3. Discussion

Transcatheter tricuspid regurgitation (TR) treatment is an emerging therapeutic modality that has gained attention in recent years. Historically, tricuspid valve surgery has been the standard treatment for significant TR. However, due to the high risk associated with open-heart surgery in many patients, the development of transcatheter therapies has provided an alternative for those who are not suitable candidates for surgery. Transcatheter tricuspid valve repair (TTVR) and transcatheter tricuspid valve replacement are the two major transcatheter therapies currently under investigation. Several studies have evaluated the safety and efficacy of transcatheter tricuspid valve therapies with promising results. 

The FORMA device consists of a spacer placed in the regurgitant orifice and a rail anchor to the endocardial surface of the right ventricle. We included five studies in this review. The sample size in the studies ranged from seven to twenty-nine patients. A multicenter study involving 19 patients with severe TR showed that the FORMA device was associated with significant improvements in TR severity, a favorable long-term safety profile, and sustained functional improvement at 2 to 3 years post-procedure [17]. The FORMA device demonstrated low rates of procedural complications. Across all studies, there were seven cases of major bleeding, three inpatient deaths, and one stroke.

MitraClip is a transcatheter device that was originally developed for the treatment of mitral regurgitation, but it has also been investigated for the treatment of TR. The device consists of a clip attached to the tricuspid valve leaflets to reduce regurgitant flow. The MitraClip for TR is still considered investigational, and early results have shown mixed outcomes. Some studies have reported significant reductions in TR severity and improved symptoms with MitraClip, while others have shown limited efficacy and a high rate of procedural complications. Further research is needed to evaluate the long-term safety and efficacy of MitraClip for TR.

The PASCAL device is a novel transcatheter device that has been developed for tricuspid regurgitation treatment. The device consists of two paddles connected by a central spacer that is placed on either side of the tricuspid valve leaflets to close the valve and prevent the backflow of blood. The Clasp II TR trial is a multicenter, randomized controlled trial evaluating patients with severe TR who are not suitable candidates for tricuspid valve surgery due to an intermediate or greater risk of mortality. Patients are currently being enrolled and randomized 1:1 to the PASCAL device with optimal medical therapy (OMT) versus OMT alone. The primary outcome is a composite endpoint including all-cause mortality, right ventricular assist device (RVAD) implantation or heart transplant, tricuspid valve intervention, heart failure hospitalizations, and quality of life improvement (measured by KCCQ score) at 24 months. Prior clinical studies evaluated the safety and efficacy of the PASCAL device for the treatment of severe TR. One study involving 30 patients with severe TR showed that the PASCAL device was associated with a significant reduction in TR severity, with 90% of patients achieving mild or less regurgitation at six months post-procedure [37]. Additionally, the study reported significant improvements in NYHA functional class and quality of life measures at six months post-procedure. It also reported significant improvements in right ventricular function and NYHA functional class at six months post-procedure. Both studies reported low rates of procedural complications, such as death, stroke, and major bleeding. Overall, the PASCAL device appears to be a safe and effective option for the treatment of tricuspid regurgitation, with the potential to improve patients’ quality of life. 

To the best of our knowledge, this is the first systematic review that assessed and compared clinical and echocardiographic outcomes of coaptation and annuloplasty devices. We included thirty studies reporting the utility of coaptation devices including TriClip, MitraClip, and PASCAL. Coaptation was the most common intervention type followed by annuloplasty then replacement. Patients selected for TTVR were elderly and at high surgical risk with multiple comorbidities. In each study, a multidisciplinary heart team assessed each patient for transcatheter valve intervention. The majority of patients had severe, massive, or torrential functional TR. Implantation through a transfemoral approach was the most common method. Common exclusion criteria were primary TR, prior tricuspid valve surgery, severe left ventricular dysfunction, large coaptation gaps, restricted leaflet mobility due to an implantable cardiac device, and severe RV dysfunction. TTVR was associated with a significant reduction in TR grade with a low rate of residual TR at 30 days. Rates of periprocedural complications including major bleeding, device-related complications, stroke, and in-hospital mortality were low regardless of device type. With regards to device preference, we presented comparative data between coaptation and annuloplasty, although our findings were limited since there were significantly more patients who underwent coaptation. Average procedural success was higher with coaptation devices, although average TR grade reduction at 30 days was higher in patients who underwent annuloplasty. Only five studies, with a small number of participants, reported the use of annuloplasty, likely due to anatomical challenges, such as anchor/suture detachment, a larger tricuspid annulus compared to mitral, and proximity to the right coronary artery.

The TRILUMINATE trial was an international, prospective, single-arm, multicenter study investigating the safety and performance of the TriClip Tricuspid Valve Repair System. Baseline echocardiography data consistent with severe TR were comparable to other studies in this review. A total of 85 patients with moderate to severe tricuspid regurgitation were included. Technical, procedural, and device success was excellent. TriClip was shown to significantly reduce TR severity by at least one grade in 91% of patients [39]. Patients experienced an improvement in functional status based on an increase in 6MWD, KCCQ score, and NYHA functional class I/II [39]. It also showed significant reverse right ventricular remodeling with regards to size and function. Other primary endpoints such as the incidence of death from any cause, tricuspid valve surgery, and the rate of hospitalization for heart failure did not differ between the groups. An important limitation of TRILUMINATE was the lack of a sham-procedure placebo control resulting in bias before patient enrollment, thus questioning the value of the positive primary endpoint based on improvement in the KCCQ. A multicenter retrospective analysis in Spain included 34 patients with severe, massive, or torrential TR [40]. The primary endpoint of TR grade reduction by one on discharge was met in 100% of patients. A total of 88% were in NYHA functional class ≤ 2 at 3 months [40].

**Figure 2:**
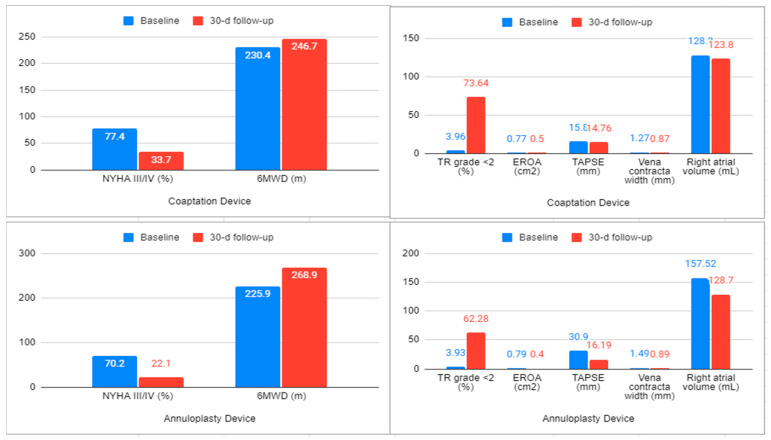
(**Upper and lower left**) NYHA functional class distribution and 6 min walking distance (6MWD) parameters of annuloplasty and coaptation devices at baseline and 30-day follow up. (**Upper and lower right**) Echocardiographic parameters at baseline and after 30-day follow-up in patients undergoing TTVR with the annuloplasty and coaptation devices.

Overall, the outcomes demonstrated excellent technical and procedural success across different TTVR platforms. The follow-up period ranged from 30 days to 1 year, although we only reported 30-day outcomes since there was limited comparable data beyond this period. A prior systematic review reported 6 month outcomes but their primary and secondary outcomes were limited to all-cause and cardiovascular mortality, heart failure hospitalization, and major adverse events [44]. There was no significant difference between baseline mean LVEF and LVEF at 30 days but most patients had a normal LVEF at baseline. Our findings demonstrated the utility of coaptation and annuloplasty devices for improvement in functional status as evident from an improvement in 6MWD and reduction in NYHA III/IV at 30 days. The number of patients in NYHA III/IV was reduced from 77.2% to 17.3% at 30 days. This is the first systematic review, to our knowledge, that included comparable echocardiographic findings from different device types (Figure 2) and demonstrated similar improvements in EROA and vena contracta width among both coaptation and annuloplasty devices. There was a reduction in right atrial volume after annuloplasty, from 157.5 to 128.7 mL. Right atrial volume in patients who underwent coaptation was 128.2 mL with a reduction to 123.8 mL. Procedural time was highest in patients who underwent annuloplasty, ranging from 188.5 min to 248 min. Procedural time with the coaptation device ranged from 58 to 170 min. Procedural time was higher with annuloplasty likely due to a lower operator experience with the Cardioband device at the time. The most common MAEs across all device types were major bleeding with 64 total events, acute kidney injury with 24 total events, and device detachment with 14 total events.

Despite the findings in our systematic review, several limitations should be addressed. Due to the nature of observational studies, selection bias was a significant limitation with device preference based on operator experience. Additionally, the majority of studies were conducted at tertiary care centers, making it difficult to translate results to centers with less experience in transcatheter interventions. Several studies had small sample sizes of less than 30 patients, indicating the early-stage utility of multiple devices. Limitations in patient characteristics affect the generalizability of the results within this review. For example, few patients with reduced LVEF were included, evident from an overall mean LVEF of 51.8%. Additionally, some studies lacked baseline echocardiographic data (as demonstrated by the gaps in Table 3), limiting our ability to compare post-interventional results among studies. Since a smaller cohort underwent annuloplasty or replacement compared to coaptation, the ability to compare outcomes among the three device types was limited.

## 4. Conclusions

Transcatheter tricuspid repair using coaptation and annuloplasty devices appears to emerge as a valuable option in the treatment of tricuspid regurgitation. Even though definitive conclusions are premature due to limited long-term outcomes, the current TTVR systems demonstrate favorable technical success in addition to symptomatic improvements. More long-term and comparative-effectiveness studies are needed to assess the long-term outcomes of these coaptation and annuloplasty devices. 

## Figures and Tables

**Table 1:** Characteristics of individual devices.

Device	Type of Device	Features
TriClip	Leaflet coaptation	Dual clip configuration, low profile delivery system, long-term durability, complementary to other devices
PASCAL	Leaflet coaptation	Clip-based system, dual leaflet grasping, hybrid design combining MitraClip and FORMA, customizable
MitraClip	Leaflet coaptation	Clip-based system, customizable, multiple clips,
FORMA	Leaflet coaptation	Annuloplasty ring, customizable
TriAlign	Direct suture annuloplasty	TV alignment, complementary to other devices
TriCinch	Ring annuloplasty	Leaflet capture, complementary to other devices
Cardioband	Ring annuloplasty	Targets annular dilation, annular reduction

**Table 2:** Baseline clinical characteristics.

Studies	N	Device Type	Age	Female (%)	STS	EUROSCORE (%)	NYHA III/IV	6MWD (m)	CABG	Prior Valve Intervention
Davidson et al. [12] (PC)	30	Annuloplasty/CB	77 ± 8	80	-	-	21 (70%)	186 ± 26.5	1 (3.3%)	13 (43.4%)
Korber et al. [13] (RC)	60	Annuloplasty/CB	76 ± 2.2	62	-	3.9 ± 1.5	49 (81.7%)	245.8 ± 125.3	-	11 (18.3%)
Nickenig et al. [14] (PC)	61	Annuloplasty/CB	78.6 ± 5.7	75	7.1 ± 5.4	6.8 ± 10.1	52 (85.2%)	-	5 (8.2%)	-
Nickenig et al. [14] (PC)	30	Annuloplasty/CB	75.2 ± 6.6	73	2.6 ± 1.6	4.1 ± 2.8	5 (18%)	-	7 (23.3%)	-
Gray et al. [15] (PC)	37	Annuloplasty/CB	77.5 ± 7.5	76	-	-	24 (64.9%)	-	2 (5.4%)	14 (37.8%)
Brener et al. [16] (PC)	444	MC, TA, TriCinch, CAVI, FORMA, CB	76.9 ± 9.1	54	-	9.9 ± 10.4	406 (91.4%)	-	-	-
Asmarats et al. [17] (PC)	19	Repair/FORMA	76 ± 9	74	-	9.2 ± 5.6	14 (95%)	256.1 ± 103.2	8 (42.1%)	14 (73.7%)
Parada et al. [18] (PC)	7	Repair/FORMA	76 ± 13	43	-	-	6 (86%)	297 ± 66	5 (71%)	4 (57%)
Gidon Perlman et al. [19] (RCT)	18	Repair/FORMA	76 ± 9.7	72	-	9.0 ± 5.7	17 (95%)	258 ± 127	13 (72%)	9 (50%)
Muntané-Carol et al. [20] (RCT)	18	Repair/FORMA, comp care	76 ± 10	72	-	9.0 ± 5.7	17 (94%)	-	-	13 (72%)
Perlman et al. [19] (RCT)	29	Repair/FORMA, US EFS	75.9 ± 8.2	66	9.1 ± 6.8	8.1 ± 5.3	25 (86%)	183 ± 96	9 (31%)	14 (48%)
Muntané-Carol et al. [21] (RCT)	29	Repair/FORMA, US EFS	76 ± 8	66	-	8.1 ± 5.3	25 (86%	-	-	0
Orban et al. [22] (RC)	50	Repair/MC	77 ± 8	40	5.6 ± 5.4	8.8 ± 6.6	50 (100%)	191 ± 124	9 (18%)	-
Lurz et al. [23] (RC)	42	Repair/MC	76.8 ± 7.3	43	4.4 ± 2.5	8.1 ± 5.7	38 (92%)	191 ± 124	5 (12%)	4 (10%)
Nickenig et al. [24] (PC)	64	Repair/MC	76.6 ± 9.6	55	4.7 ± 4.6	27.8 ± 16.7	60 (97%)	243.5 ± 28.5	26 (40%)	21 (33%)
Besler et al. [25] (RC)	117	Repair/MC	79 ± 1.8	44	5.3 ± 1.1	6.3 ± 1.7	113 (97%)	177.4 ± 103.0	19 (16%)	23 (20%)
Michael Mehr et al. [26] (RC)	249	Repair/MC	77 ± 9	51	-	6.4 ± 2.5	238 (95.6%)	-	-	27 (10.8%)
Daniel Braun et al. [27] (PC)	18	Repair/MC	78 ± 7	44	5 ± 3	10.0 ± 8	19 (100%)	261 ± 110	4 (22%)	-
Stocker et al. [28] (PC)	236	Repair/MC and PC	78 ± 4	53	-	5.1 ± 1.7	209 (89%)	229 ± 48.8	-	-
Faeez M Ali et al. [29] (PC)	20	Repair/MC NTR	72.2 ± 5.8	70	6.1 ± 1.6	8.0 ± 1.9	19 (95%)	-	3 (15%)	-
Cai et al. [30] (RC)	53	Repair/MC NTR	74.8 ± 11.1	59	-	-	43 (93.5%)	279.0 ± 144.7	11 (20.8%)	-
Otto et al. [31] (PC)	20	Repair/MC NTR/XTR	78.6 ± 8.3	50	8.8 ± 4.3	9.1 ± 7.7	18 (90%)	-	-	3 (15%)
Faeez M Ali et al. [29] (PC)	20	Repair/MC XTR	78.1 ± 4.3		10.5 ± 3.1	13 ± 4.5	20 (100%)	-	6 (30%)	-
Daniel Braun et al. [32] (RC)	31	Repair/MC XTR	77 ± 5		5 ± 5	-	28 (90%)		-	-
Friedrich Ruf et al. [33] (RC)	50	Repair/MC XTR	80 ± 2	58	-	-	49 (98%)	-	-	1 (2%)
Sugiura et al. [34] (RC)	22	Repair/MC-XTR	79 ± 5	64	-	7.4 ± 1.4	20 (91%)	-	-	16 (73%)
David Planer et al. [35] (RCT)	7	Repair/MISTRAL	73.1 ± 7.4	57	5.3 ± 0.6	4.0 (3.6–6.8)	7 (100%)	-	-	3 (43%)
Kodali et al. [36] (RCT)	34	Repair/PC	76.3 ± 10.4	53	7.3 ± 4.7	5.3 ± 5.2	27 (79%)	180 ± 101	10 (29.4%)	-
Kitamura et al. [37] (PC)	30	Repair/PC	77 ± 6	57	4.4 ± 2.8	5.7 ± 5.2	27 (90%)	275 ± 122	3 (10%)	-
Volz et al. [38] (RC)	11	Repair/PC	71 ± 9	27	-	5.5 ± 3.7	11 (100%)	-	-	5 (45%)
Sugiura et al. [34] (RC)	22	Repair/PC	79 ± 5	64	-	7.5 [4.8, 12.1]	21 (96%)	-	-	14 (64)
Philipp Lurz et al. [39] (RCT)	85	Repair/TC	77.8 ± 7.9	66	-	8.7 ± 10.7	45 (69%)	272.3 ± 15.6	-	-
Xavier Freixa et al. [40] (RC)	34	Repair/TC	75.5 ± 2.5	74	3.73 ± 0.7	4.0 [2.3–5.7]	25 (76%)	-	-	12 (35%)
Sorajja et al. [41] (RCT)	175	Repair/TC	78 ± 7.4	56	-	-	104 (59.4%)	240.5 ± 117.1	31 (17.7%)	70 (40%)
F Meijerink et al. [42] (RC)	21	Repair/TC/MC	78 ± 6.8	66	-	-	20 (95%)	-	1 (5%)	7(9%)
Cepas-Guillen et al. [43] (PC)	28	Repair/TC/MC	76.5 ± 2.5	89	-	3.9 (2–7)	25 (88%)	272.3 ± 15.6	-	-
Total/Weighted Avg	-	(Annuloplasty devices)	76.9 (1.3)	71% (7.1%)	5.6 (2.1)	5.1 (1.4)	70.2% (24.5%)	225.9 (28.3%)	10.7% (7.5%)	26.7% (11.9%)
Total/Weighted Avg	-	(Coaptation devices)	77.2 (1.2)	56.5% (10.4%)	5.7 (1.7)	8.4 (4.37)	77.4% (28.1%)	230.4 (33.2%)	21.9% (13.2%)	28.1% (19.2%)
Total	2273	-	77.2 (1.2)	58.4% (11.8%)	5.9 (1.9)	8.1 (4.24)	77.2% (27.6%)	230.7 (32.8%)	20.3% (12.8%)	27.9% (18.7%)

TriClip = TC, MitraClip = MC, TriAlign = TA, PASCAL = P; Cardioband = CB, TriCinch = TCI; prospective cohort = PC; retrospective cohort = RC; randomized controlled trial = RCT. (-) indicates no data available. EuroSCORE, Logistic European System for Cardiac Operative Risk Evaluation; STS, Society of Thoracic Surgery risk score.

**Table 3:** Baseline echocardiographic characteristics.

Studies	Device Type	Tricuspid Regurgitation n (%)			Severe TR; n (%)	Baseline				
		Degenerative	Functional	Mixed		EROA	Reg. Volume	LVEF (%)	RA Vol (mL)	TAPSE
Davidson et al. [12]	annuloplasty/CB	-	30 (100%)	-	30 (100%)	0.8 ± 0.4		58.4 ± 5.6	134.6 ± 41.6	
Korber et al. [13]	annuloplasty/CB	-	60 (100%)	-	60 (100%)			55.9 ± 8.6		17.9 ± 4.3
Nickenig et al. [14]	annuloplasty/CB	-	61 (100%)	-	57 (94%)	0.8 ± 0.5	-	53.3 ± 7.6	168.8 ± 69.3	-
Nickenig et al. [44]	annuloplasty/CB	-	30 (100%)	-	19 (76%)	0.8 ± 0.5	79.4 ± 29.6	57.2 ± 10.5	-	-
Gray et al. [15]	annuloplasty/CB		36 (97.3%)	1 (2.7%)	37 (100%)			57.6 ± 5.7		
Brener et al. [16]	MC, TA, TriCinch, CAVI, FORMA, CB	26 (5.9%)	393 (88.5%)	20 (4.5%)	434 (97.7%)	0.7 ± 0.5	-	-	-	16.4 ± 4.6
Asmarats et al. [17]	repair/FORMA	-	19 (100%)	-	18 (95%)	0.9 ± 0.6	-	60 ± 9	164.9 ± 90.5	15.3 ± 4.6
Parada et al. [18]	repair/FORMA	-	7 (100%)	-	7 (100%)	-	-	56 ± 5	-	16.5 ± 4.2
Gidon Perlman et al. [19]	repair/FORMA	-	18 (100%)	-	-	1.0 ± 0.6	-	59 ± 9	143 ± 59	14.7 ± 5.4
Muntané-Carol et al. [20]	repair/FORMA, comp care	-	-	-	-	-	-	-	-	-
Perlman et al. [19]	repair/FORMA, US EFS	-	25 (100%)	-	-	1.1 ± 0.6		55.9 ± 13.8		14.0 ± 4.0
Muntané-Carol et al. [21]	repair/FORMA, US EFS trial	-	-	-	-	2.1 ± 1.8		-	-	-
Orban et al. [22]	repair/MC	1 (2%)	49 (98%)	-	49 (98%)			46 ± 16		16 ± 4.1
Lurz et al. [23]	repair/MC	1 (3%)	41 (97%)	-	37 (91%)		48 ± 30, * 50 ± 23 **	39 ± 18, * 56 ± 12 ***	71 ± 24, * 80 ± 30 ***	16 ± 5, * 16 ± 3 ***
Nickenig et al. [24]	repair/MC	5 (8%)	56 (88%)	3 (4%)	56 (88%)	0.9 ± 0.4	59.9 ± 18.4	46.9 ± 13.9		16.9 ± 5.8
Besler et al. [25]	repair/MC	4 (3%)	117 (97%)	-	-					
Michael Mehr et al. [26]	repair/MC	11 (4.4%)	223 (89.6%)	7 (2.8%)	241 (96.8%)	0.70 ± 0.5	-	49 ± 14	106.5 ± 74.6	15.8 ± 4.3
Daniel Braun et al. [27]	repair/MC	-	31 (100%)	-	16 (88.3%)	-	-	-	-	16 ± 3
Stocker et al. [28]	repair/MC and PC	-	-	-	236 (100%)	-	-	55 ± 2.5	-	17 ± 1.8
Faeez M Ali et al. [29]	repair/MC NTR	1 (5%)	17 (85%)	2 (10%)	20 (100%)	0.7 ± 0.6	56.4 ± 5.9	49.1 ± 6.2	113.3 ± 16.1	17.9 ± 2.1
Cai et al. [30]	repair/MC NTR	5 (9.4%)	47 (88.7%)	-	53 (100%)	-	-	49.7 ± 16.6	-	15.6 ± 3.4
Otto et al. [31]	repair/MC NTR/XTR	5 (25%)	14 (70%)	1 (5%)	20 (100%)	0.7 ± 0.3		54.3 ± 14.6		
Faeez M Ali et al. [29]	repair/MC XTR	0	17 (85%)	3 (15%)	20 (100%)	0.8 ± 0.2	61.2 ± 8.6	46.4 ± 6.5	179.9 ± 71.8	16.7 ± 2
Daniel Braun et al. [32]	repair/MC XTR	-	31 (100%)	-	29 (94%)	-	-	-	-	-
Friedrich Ruf et al. [33]	repair/MC XTR	-	-	-	43 (86%)	-	-	55.5 ± 1.3	144.1 ± 28.6 ^B^	15.5 ± 2 ^C^
Sugiura et al. [34]	repair/MC-XTR	-	-	-	16 (73%)	0.7 ± 0.1	53.6 ± 4.6	56.9 ± 1.0		18.0 ± 1.5
David Planer et al. [35]	repair/MISTRAL	-	7 (100%)	-	7 (100%)	0.5 ± 0.1	49.4 ± 3.1	58.0 ± 1.4		16.0 ± 1.0
Kodali et al. [36]	repair/PC	2 (6.1%)	29 (87.9%)	2 (6.1%)	33 (100%)			57.4 ± 7.0		15.3 ± 4.7
Kitamura et al. [37]	repair/PC	3 (10%)	25 (83%)	2 (7%)	30 (100%)	-	-	-		16.8 ± 4.1
Volz et al. [38]	repair/PC	-	11 (100%)	-	11 (100%)	-	-	43 ± 15	-	16.0 ± 4.0
Sugiura et al. [34]	repair/PC	-	-	-	16 (73%)	0.8 ± 0.2	51.8 ± 5.8	57.0 ± 2.2		16.5 ± 1.6
Philipp Lurz et al. [39]	repair/TC	10 (12%)	71 (84%)	3 (4%)	57 (92%)	0.65 ± 0.1	52.2 ± 2.4	-	129 ± 5.84	14.4 ± 0.3
Xavier Freixa et al. [40]	repair/TC	1 (3%)	27 (79%)	6 (18%)	34 (100%)	-	-	57.5 ± 1.5	28 ± 3.1	18 ± 1.3
Sorajja et al. [41]	repair/TC	-	165 (94.8)	-	136 (97%)	0.7 ± 0.3	53.9 ± 18.8	59.3 ± 9.3	143.2 ± 85.4	
F Meijerink et al. [42]	repair/TC/MC	-	21 (100%)	-	21 (100%)	-	-	-	134 (70–678)	13 ± 4.5
Cepas-Guillen et al. [43]	repair/TC/MC	1 (3%)	26 (94%)	1 (3%)	28 (100%)	-	-	59 ± 1.3	-	-
Weighted avg (annuloplasty)					96.07	0.79	79.4	55.98	157.52	30.87
Weighted avg (coaptation)					96.24	0.77	54.27	53.17	128.16	15.82

(-) indicates no data available. Effective regurgitant orifice area (EROA); right atrium (RA); tricuspid annular plane systolic excursion (TAPSE); * (TV and MV), ** (MV), *** (TV); ^B^ = (n = 39); ^C^ = (41).

**Table 4:** Procedural and midterm clinical outcomes.

Studies	Device Type	Success; n (%)		TR Gr Change ≥ 1; n (%)		30-Day Outcomes							
		Technical	Procedural	Post-Op	30 Day	6MWD (m)	TR ≤ 2 Grade	NYHA III/IV; n(%)	EROA	Reg Vol	LVEF%	RAV	TAPSE
Davidson et al. [12]	annuloplasty/CB	28 (93%)	-	-	24 (85%)	246 ± 125	12 (45%)	7 (25%)	0.55 ± 0.41	-	-	105.7 ± 42.5	39.5 ± 7.4
Korber et al. [13]	annuloplasty/CB	58 (97%)	27 (45%)	-	41 (73%)	-	67 (40%)	18 (80%)	-	-	54.8 ± 6.7	-	-
Nickenig et al. [14]	annuloplasty/CB	-	26 (84%)	40 (78%)	33 (85%)	-	23 (59%)	13 (26%)	0.34 ± 0.27	-	54.8 ± 8.2	140 ± 66	15.0 ± 4.0
Nickenig et al. [44]	annuloplasty/CB	30(100%)	-	-	-	292 ± 123	23 (76%)	5 (18%)	0.39 ± 0.32	43.7 ± 34.1	57.7 ± 8.0	-	-
Gray et al. [15]	annuloplasty/CB	34 (92%)	31 (82.6%)	-	-	-		-	0.8 ± 0.4	-	57.6 ± 5.7	141.1 ± 46.3	17.0 ± 3.0
Brener et al. [16]	MC, TA, TriCinch, CAVI, FORMA, CB	-	-	-	-	-	227 (71%)	102 (31%)	-	-	-	-	15.6 ± 4.4
Asmarats et al. [17]	repair/FORMA	-	-	-	-	340 ± 87	-	6 (35%)	-	-	-	139 ± 10	16 ± 1.5
Campelo-Parada et al. [18]	repair/FORMA	7 (100%)	-	7 (100%)	-	326 ± 74	-	-	-	-	57 ± 4	-	18.5 ± 5.3
Perlman et al. [19]	repair/FORMA	16 (89%)	-	-	-	327 ± 93	-	1 (6%)	0.4 ± 0.3	-	61 ± 9	-	14.0 ± 0.3
Muntané-Carol et al. [21,23]	repair/FORMA, comp care	16 (89%)	-	-	-	84	-	4 (21%)	-	-	-	-	-
Gidon Perlman et al. [19]	repair/FORMA, EFS	27 (93%)	-	-	-	256 ± 103	-	8 (28%)	0.6 ± 0.4	-	58.6 ± 12.9	-	15.0 ± 4.0
	repair/FORMA, comp	16 (88%)	-	-	-	39	-	1(6%)	1.1 ± 0.9	-	-	-	-
Orban et al. [22]	repair/MC	47 (94%)	-	46 (92%)	-	238 ± 132	34 (74%)	16 (35%)	-	-	-	-	-
Lurz et al. [23]	repair/MC	-	79 (83%)	35 (83%)	-	-	29 (82%)	5 (20%) *; 1 (10%) ^$^	0.3 ± 0.3 * 0.2 ± 0.1 ^$^	27 ± 24 *, 16 ± 8 ^$^	39 ± 17 * 53 ± 13 ^$^	67 ± 31 *, 64 ± 2 9 ^$^	17 ± 5 *, 17 ± 4 ^$^
Nickenig et al. [24]	repair/MC	62 (97%)	-	58 (91%)	-	194 ± 116	52 (81%)	16 ^&^ (41%), 15 ^%^ (75%) ^%^	0.4 ± 0.2 ^&^, 0.4 ± 0.1 ^%^	30.8 ± 6.9 ^&^ 29.9 ± 8.2 ^%^	-	-	-
Besler et al. [25]	repair/MC	112 (96%)	95 (81%)	95 (81%)	-	-	-	-	-	-	-	-	-
Mehr et al. [26]	repair/MC	239 (96%)	192 (77%)	222 (89%)	-	-	-	-	-	-	-	-	-
Braun et al. [27]	repair/MC	-	18 (100%)	-	16 (89%)	267 ± 108	12 (65%)	9 (30%)	0.28 ± 0.21	29 ± 19	46 ± 17	-	15.0 ± 4.0
Stocker et al. [28]	repair/MC and PC	-	-	192 (84%)	-	-	-	-	-	-	-	-	-
Ali et al. [29]	repair/MC NTR	-	-	-	-	-	-	6 (30%)	-	-	-	112.7 ± 19.7	15.8 ± 1.4
Cai et al. [30]	repair/MC NTR	-	40 (77.3%)	-	-	-	-	-	-	-	-	-	-
Otto et al. [31]	repair/MC NTR/XTR	15 (75%)	-	-	-	-	-	13 (67%)	-	-	-	-	-
Ali et al. [29]	repair/MC XTR	-	-	-	-	-	-	3 (15%)	-	-	-	171.5 ± 73.3	16.6 ± 2.3
Braun et al. [32]	repair/MC XTR	17 (87%)	-	-	-	-	12 (69%)	10 (31%)	-	-	-	-	-
Ruf et al. [33]	repair/MC XTR	50 (100%)	-	-	-	312 ± 21x	-	22 (44%)	-	-	-	149.9 ± 31.1	13.3 ± 1.7
Sugiura et al. [34]	repair/MC-XTR	-	-	-	-	224 ± 104	15 (68%)	4 (18%)	-	-	-	-	-
Planer et al. [35]	repair/MISTRAL	-	7 (100%)	7 (100%)	-	288 ± 37.3	-	0%	0.15 ± 0.02	19.7 ± 2.9	-	-	20.0 ± 0.8
Kodali et al. [36]	repair/PC	-	24 (80%)		25 (85%)	251 ± 100	20 (70%)	3 (11%)	-	-	-	-	-
Kitamura et al. [37]	repair/PC	25 (83%)	-	-	-	328 ± 115	24 (81%)	2 (10%)	-	-	-	-	15.0 ± 3.5
Volz et al. [38]	repair/PC	-	-	-	-	-		-	-	-	44 ± 14	-	15.0 ± 4.0
Sugiura et al. [34]	repair/PC	21 (96%)	-	21 (96%)	-	224 ± 104	11 (50%)	15 (7%)	-	-	-	-	-
Lurz et al. [39]	repair/TC	-	-	-	25 (60%)	297 ± 13	25 (60%)	12 (18%)	0.40 ± 0.03	34.8 ± 2.9	-	117 ± 6.03	14.9 ± 0.3
Freixa et al. [40]	repair/TC	-	-	34 (100%)	-	-	-		-	-	-	-	-
Sorajja et al. [41]	repair/TC	170 (99%)	141 (87%)	-	-	-	156 (87%)	-	-	-	-	-	-
F Meijerink et al. [42]	repair/TC/MC	18 (86%)	16 (76%)	17 (81%)	-	-	-	6 (29%)	-	-	-	-	-
Cepas-Guillen et al. [43]	repair/TC/MC	-	-	-	-	-	-		-	-	-	-	-
Weighted Avg (Annuloplasty)		96.7% (2.4%)	64.6% (19.5%)	-	80.3% (5.8%)	268.9 (23.3)	62.3% (9.7%)	22.1% (3.6%)	0.4 (0.1)	N/A	55.4 (1.2)	128.7 (16.2)	16.2
Weighted Avg (Coaptation)		94.8% (4.8%)	81.4% (5.4%)	87.2% (4.9%)	68.1% (12.0%)	246.67 (76.4)	73.6% (8.5%)	33.7% (18.3%)	0.5 (0.2)	30.9 (3.7)	51.1 (8.8)	123.8 (29.5)	14.8

(-) indicates no data available. * (TV and MV), (MV), ^$^ (TV); ^&^ TR Clip; ^%^ TR and mitral clip.

**Table 5:** Major adverse events (MAEs) of included studies.

Studies	Procedural Time (mins)	Major Bleeding	Device Detachment	Device Embolization	Device Thrombosis	Stroke	AKI	Access Site Complications	Conversion to Surgery	In-Hospital Mortality
Charles J Davidson et al.	217 ± 80	7	-	-	-	0	0	2	0	0
Korber et al. [13]	248 ± 77	6	-	-	-	0	7	1	2	2
Nickenig et al. [14]		-	-	-	-	0	2	4		1
Nickenig et al. [44]			-	-	-	-	-	-	-	-
Gray et al. [15]	188.5 ± 88.8		-	-	-	-	-	-	-	-
Philipp Lurz et al. (TRILUMINATE trial) 1 year [39]	-	-	-	-	-	-	-	-	-	-
Lluis Asmarats et al. [17]	-	2	-	-	1	1	0	0	1	1
Francisco Campelo-Parada et al. [18]	122 ± 34	0	-	-	-	-	1	1	0	0
Gidon Perlman et al. [19]	-	1	-	-	-	0	0	0	-	0
Guillem Muntané-Carol et al. [20]	-	-	1	-	-	-	-	-	-	-
Gidon Perlman et al. [19]	-	4				0	3			2
Guillem Muntané-Carol et al. [21]	-	-	2		-	-	-	-	-	-
Mathias Orban et al. [22]	-	-	-	-	-	-	-	-	2	-
Philipp Lurz et al. [23]	102.5 ± 47.2	-	2	0		-	-	-	-	0
Georg Nickenig et al. [24]	143.4 ± 91.6	3		0		0	2	0	0	3
Christian Besler et al. [25]	-	1	-	-	-	1	-	-	-	2
Michael Mehr et al. (1 year) [26]	136 ± 62	15	-	-	-	2	9	-	1	7
Daniel Braun et al. [27]						0				
Stocker et al. [28]	-	-	-	-	-	-		-	-	-
Cai et al. [29]	-	-	-	-	-	0	-	-	-	0
Faeez M Ali et al. (MC NTR) [30]	-	0		0		0	0	-	0	0
Otto et al. [31]	170.0 ± 75.8	2	-	-	-	-	3	-	0	1
Faeez M Ali et al. (MC XTR) [29]	-	0	-	0	-	0	0	-	0	0
Daniel Braun et al [32]	-	-	4		-	-	-	-	-	-
Tobias Friedrich Ruf et al. [33]	-	-	3	-	-	-		-	-	3
Sugiura et al. (MC-XTR) [34]	67.0 ± 13.1	1	-	-	-	0	-	-	0	0
David Planer et al. [35]		-	-	-	-	-	-	-	-	-
Kodali et al. [36]	167.7	2				0	0	0	0	0
Mitsunobu Kitamura et al. [37]	-	-	-	-	-	-	-	-	-	-
Volz et a. [38]	-	-	-	-	-	-	-	-	-	-
Sugiura et al. (PC) [34]	62.0 ± 37.5	2	-	-	-	0		-	0	0
	130	0	1 (Partial)	-	-	0		0	0	0
Sorajja et al. [41]	151.0 ± 71.7	9		0	0	-	-	-	-	0
F Meijerink et al. [42]		5	2	0	0	0	0	0	2	0
Pedro Luis Cepas-Guillen et al. [43]	130		3 (partial)	0	0	0	-	1	0	0
Michael I Brener et al. [16]	-	-	-	-	-	5	-	-	4	10
Total Number of Events		67	14	0	1	9	27	9	12	32

(-) indicates no data available.

## Supplementary Materials

The following supporting information can be downloaded at: https://www.mdpi.com/article/10.3390/jcm13216531/s1, Table S1: Exclusion criteria for devices reported. Table S2: Baseline clinical characteristics.

## Abbreviations

Tricuspid regurgitation (TR), transcatheter tricuspid valve intervention (TTVI), tricuspid annular plane systolic excursion (TAPSE), European System for Cardiac Operative Risk Evaluation (EuroSCORE).
