# Supplementary material for: Transcatheter Repair of Tricuspid Valve Regurgitation: A Systematic Review
Source: J Clin Med. 2024 Oct 30;13(21):6531. doi: 10.3390/jcm13216531 (PMC11546873; doi:10.3390/jcm13216531)
Supplement: Supplementary file 1 [file jcm-13-06531-s001.zip › jcm-3214677-supplementary.pdf]

Table S1. Exclusion criteria for devices reported.

Devices	Exclusion Criteria
Cardioband	Tricuspid valve anatomy precluding proper device deployment and function including primary tricuspid valve disease, previous tricuspid valve repair or replacement, the presence of a transtricuspid pacemaker or defibrillator, LVEF <25%, severe right ventricular dysfunction, eGFR ≤25 mL/min/1.73 m^2 or chronic dialysis, chronic anemia, pulmonary arterial systolic pressure >70 mmHg, or tricuspid proximal isovelocity surface area (PISA) effective regurgitant orifice area (EROA) ≥2.0 cm^2 [12–14].
FORMA	Primary TR, prior tricuspid valve surgery, severe concomitant valve disease, severe left ventricular dysfunction, or pacemaker leads that are adherent to the valve leaflet [18,20].
MitraClip	Only moderate TR and tricuspid annulus <40 mm on TEE; large coaptation gaps impossible to bridge with the device; any mitral or tricuspid valve stenosis; poor echocardiographic visibility during screening TEE; patients recommended by an interdisciplinary heart team to undergo a surgical procedure; any transtricuspid RV lead hindering leaflet adaption and thus the predominant reason for TR; severe aortic stenosis or patients undergoing a transcatheter aortic valve replacement procedure for this reason; patients undergoing heart transplantation; systolic pulmonary arterial pressure >60 mm Hg; or severe coaptation defect (>2 cm) [14,15,24].
TriClip	Asymptomatic. pulmonary artery systolic pressure of greater than 70 mm Hg [45,46].
PASCAL	A systolic pulmonary artery (PA) pressure > 60 mmHg was defined as an exclusion criterion to avoid early RV failure [41].
MISTRAL	Tricuspid stenosis, the calcification of the tricuspid subvalvular apparatus or the chordae tendineae, intracardiac mass, a history of endocarditis or active infection including endocarditis, and inability to perform transesophageal echocardiography (TEE) [38].

Table S2. Baseline clinical characteristics.

Studies	N	Device Type	COPD	CAD	DM	HTN	MI	Stroke	CKD
Davidson et al. [12]	30	annuloplasty/CB	-	4 (13.3%)	9 (30.0%)	18 (62.1%)	4 (13.3%)	2 (6.7%)	11 (36.7%)
Korber et al. [13]	60	annuloplasty/CB	17 (28.3%)	26 (43.4%)	18 (30%)	-	6 (10%)	10 (16.7%)	21 (35%)

Nickenig et al. [14]	61	annuloplasty/CB	-	7 (11.9%)	17 (27.9%)	51 (85.0%)	6 (10.3%)	8 (13.6%)	26 (42.6%)
Nickenig et al. [44]	30	annuloplasty/CB	-	11 (36.7%)	8 (26.7%)	24 (80.0%)	-	5 (16.7%)	16 (53.3%)
Gray et al. [15]	37	annuloplasty/CB	-	4 (10.8%)	10 (27.0%)	26 (70.3%)	4 (10.8%)	2 (5.4%)	14 (37.8%)
Brener et al. [16]	444	MC, TA, TriCinch, CAVI, FORMA, CB	102 (22.9%)	-	122 (28.1%)	-	68 (15.4%)	-	-
Asmarats et al. [17]	19	repair/FORMA	5 (26.3%)	-	2 (10.5%)	16 (84.2%)	-	2 (11.1%)	15 (78.9%)
Parada et al. [18]	7	repair/FORMA	1 (14%)	6 (86%)	-	-	-	-	6 (86%)
Gidon Perlman et al [19]	18	repair/FORMA	5 (28%)	10 (56%)	2 (11%)	16 (89%)	-	2 (11%)	-
Muntané-Carol et al. [20]	18	repair/FORMA, comp care	-	-	-	-	-	-	-
Perlman et al. [19]	29	repair/FORMA, US EFS	7 (24%)	16 (55%)	-	-	-	11 (38%)	-
Muntané-Carol et al. [21]	29	repair/FORMA, US EFS trial	-	-	-	-	-	-	-
Orban et al. [22]	50	repair/MC	12 (24%)	16 (32%)	-	-	-	-	45 (90%)
Lurz et al. [23]	42	repair/MC	9 (21%)	16 (38%)	22 (52%)	40 (95%)	4 (10%)	-	-
Nickenig et al. [24]	64	repair/MC	18 (28%)	25 (39%)	19 (30%)	47 (73%)	14 (22%)	-	-
Besler et al. [25]	117	repair/MC	23 (20%)	37 (32%)	-	-	-	-	-
Michael Mehr et al. [26]	249	repair/MC	62 (24.9%)	103 (41.4%)	73 (29.4%)	-	45 (18.1%)	103 (41.4%)	17 (6.8%)
Daniel Braun et al. [27]	18	repair/MC	3 (17%)	-	-	-	-	-	-
Stocker et al. [28]	236	repair/MC and PC	39 (19%)	96 (43%)	60 (27%)	-	-	-	-
Faeez M Ali et al. [29]	20	repair/MC NTR	2 (10%)	4 (20%)	8 (40%)	10 (50%)	-	2 (10%)	-
Cai et al. [30]	53	repair/MC NTR	6 (11.3%)	24 (45.3%)	16 (30.2%)	36 (67.9%)	13 (24.5%)	11 (20.8%)	20 (37.8%)
Otto et al. [31]	20	repair/MC NTR/XTR	6 (30%)	5 (25%)	6 (30%)	-	-	-	2 (10%)

Faeez M Ali et al. [29]	20	repair/MC XTR	4 (20%)	9 (45%)	7 (35%)	16 (80%)	-	2 (10%)	-
Daniel Braun et al. [32]	31	repair/MC XTR	-	-	-	-	-	-	-
Friedrich Ruf et al. [33]	50	repair/MC XTR	-	-	9 (18%)	39 (78%)	4 (8%)	-	23 (46%)
Sugiura et al. [34]	22	repair/MC-XTR	3 (14%)	11 (50%)	5 (23%)	19 (86%)	-	-	-
David Planer et al. [35]	7	repair/MISTRAL	-	-	-	-	-	-	-
Kodali et al. [36]	34	repair/PC	9 (26.5%)	11 (32.4%)	4 (11.8%)	32 (94.1%)	3 (8.8%)	9 (26.4%)	15 (44.1%)
Kitamura et al. [37]	30	repair/PC	6 (20%)	9 (30%)	-	-	0(0)	-	21 (70%)
Volz et al. [38]	11	repair/PC	4 (36%)	8 (73%)	-	-	-	-	-
Sugiura et al. [34]	22	repair/PC	7 (32%)	13 (59%)	4 (18%)	18 (82%)	-	-	-
Philipp Lurz et al. [39]	85	repair/TC	-	-	19 (22%)	73 (86%)	15 (18%)	-	39 (46%)
Xavier Freixa et al. [40]	34	repair/TC	5 (15%)	2 (6%)	7 (20%)	19 (56%)	-	6 (18%)	14 (41%)
Sorajja et al. [41]	175	repair/TC	19 (10.9%)	26 (14.9%)	28 (16.0%)	142 (81.1%)		11 (6.3%)	62 (35.4%)
F Meijerink et al. [42]	21	repair/TC/MC	1 (5%)	1 (5%)	4 (19%)	-	-	-	-
Cepas-Guillen et al. [43]	28	repair/TC/MC	7 (25%)	2 (7%)	5 (18%)	17 (61%)	-	4 (14%)	-
Total/Weighted Avg (Annuloplasty devices)			N/A	26.5% (14.7%)	28.7% (1.3%)	76.0 (9.8%)	10.6% (1.4%)	13.8% (3.6%)	40.9% (6.4%)
Total/Weighted Avg (Coaptation devices)			20.5% (5.7%)	33.9% (14.1%)	25.5% (7.0%)	78.3% (10.7%)	15.6% (4.6%)	23.0% (15.3%)	34.8% (23.7%)
Total	2273		20.5% (5.9%)	32.9% (14.2%)	25.9% (6.7%)	77.6% (10.5%)	14.9% (4.8%)	21.3% (13.8%)	36.9% (21.8%)

(-) indicates no data available. CAD, coronary artery disease; HTN, hypertension; MI, myocardial infarction; CKD, chronic kidney disease.
